# Insulin-Like Growth Factor I Prevents Cellular Aging via Activation of Mitophagy

**DOI:** 10.1155/2020/4939310

**Published:** 2020-08-01

**Authors:** Xuwei Hou, Zhaohui Li, Yusuke Higashi, Patrice Delafontaine, Sergiy Sukhanov

**Affiliations:** ^1^Department of Medicine, School of Medicine, University of Missouri-Columbia, Columbia 65201, MO, USA; ^2^Heart and Vascular Institute, Tulane University Health Sciences Center, New Orleans 70112, LA, USA

## Abstract

Mitochondrial dysfunction is a hallmark of cellular aging. Mitophagy is a critical mitochondrial quality control mechanism that removes dysfunctional mitochondria and contributes to cell survival. Insulin-like growth factor 1 (IGF-1) promotes survival of smooth muscle cells (SMCs), but its potential effect on cellular aging is unknown yet. We found that IGF-1 decreased cell senescence, prevented DNA telomere shortening, increased mitochondrial membrane potential, activated cytochrome C oxidase, and reduced mitochondrial DNA damage in long-term cultured (aged) aortic SMC, suggesting an antiaging effect. IGF-1 increased mitophagy in aged cells, and this was associated with decreased expression of cyclin-dependent kinase inhibitors p16 and p21 and elevated levels of Nrf2 and Sirt3, regulators of mitophagy and mitochondrial biogenesis. SiRNA-induced inhibition of either Nrf2 or Sirt3 blocked IGF-1-induced upregulation of mitophagy, suggesting that the Nrf2/Sirt3 pathway was required for IGF-1's effect on mitophagy. PINK1 is a master regulator of mitophagy. PINK1 silencing suppressed mitophagy and inhibited IGF-1-induced antiaging effects in aged SMC, consistent with an essential role of mitophagy in IGF-1's effect on cellular aging. Thus, IGF-1 inhibited cellular aging via Nrf2/Sirt3-dependent activation of mitophagy. Our data suggest that activation of IGF-1 signaling is a novel potential strategy to activate mitophagy and slow cellular aging.

## 1. Introduction

Cellular aging is a complex biological process, which includes irreversible arrest of cell proliferation (cell senescence) and development of mitochondrial dysfunction [1, 2]. Aging-induced alterations at the cellular level are main driving forces leading to tissue-specific aging and aging of the entire organism [3]. Mitochondria play vital roles in regulation of bioenergetics and metabolic responses. Aging-induced damage of mitochondria in vascular smooth muscle cells (SMCs) inhibits SMC contractility and contributes to increased arterial stiffness and progression of aging-associated vascular pathologies [4]. To overcome loss in mitochondrial function, SMC, as well as other cells, rely on stimulation of mitochondrial biogenesis and activation of mitophagy, a tightly regulated mechanism to remove dysfunctional mitochondria. However, the efficiency of mitochondrial biogenesis and mitophagy declines with age, leading to progressive accumulation of dysfunctional mitochondria and eventual loss of SMC function and cell death [5]. Therefore, activation of mitophagy in aged cells may serve as a potential therapeutic approach to slow cellular aging and improve age-associated pathologies [5].

Insulin-like growth factor 1 (IGF-1) is an endocrine and autocrine/paracrine growth factor that is expressed by almost all cells including vascular SMC and that has major effects on cell growth, differentiation, and migration [6]. Several reports indicate that IGF-1 preserves mitochondrial function *in vitro* and *in vivo* [7–9]. Thus, cancer cell viability was dependent on IGF-1 stimulation of mitochondrial biogenesis and mitophagy [10]. In vivo studies in invertebrates (nematode and *Drosophila melanogaster*) show that inhibition of insulin/IGF-1 signaling pathway increased longevity [11]. There are conflicting reports of effect of the IGF-1 receptor heterozygosity on mice longevity [12, 13], and also, the results are contradictory in humans [14]. The potential effect of IGF-1 on cellular aging is unknown. Considering IGF-1-induced restoration of mitochondrial biogenesis, we hypothesized that IGF-1 may attenuate cellular aging of SMCs. We found that IGF-1 restored mitochondrial membrane potential (MMP) and cytochrome C oxidase activity via the nuclear respiratory factor 2 (Nrf2)/sirtuin-3 (Sirt3) axis in long-term cultured SMC, and this mitochondrial protection was associated with activation of mitophagy. Our report suggests a therapeutic potential for IGF-1 in stimulating mitophagy, thereby slowing cellular aging.

## 2. Materials and Methods

### 2.1. Cells

SMCs were isolated from mice with smooth muscle 22*α* promoter-driven IGF-1 receptor deficiency (IGF-1R-KO SMC) and from control mice (Con SMC) [15]. Mice *aortae* were cleaned of adventitial fat tissue, opened, and minced into small pieces. The pieces of *aortae* were placed on culture dishes with DMEM/F12 media (ATCC) and supplemented with 10% FBS, 4 mmol/L *L*-glutamine, 100 U/mL penicillin, and 100 *μ*g/mL streptomycin. Aortic SMCs migrated and grew out of the minced aorta were trypsinized and passaged into T75 cell culture flasks. Cell identity was confirmed by staining for SMC markers: calponin and smooth muscle *α*-actin (*α*SMA). More than 90% cells were double positive for calponin and *α*SMA [15].

To investigate a potential effect of IGF-1 on cellular aging, we performed experiments with cultured short-term (passage 5, P5) and long-term (passage 20, P20) SMC. Both P5 and P20 SMCs were cultured in complete cell media supplemented with 10% FBS, *L*-glutamine, and antibiotics. Since complete cell culture media contains IGF-1, we used serum-free media for short-term (12 hrs) and long-term (24 and 72 hrs) cell treatment with IGF-1. For these experiments, cells were cultured in DMEM supplemented with 100 U/mL penicillin, and IGF-1 was administrated for 12, 24, and 72 hrs.

### 2.2. Beta-Galactosidase Staining and Activity

SMCs were seeded at a density of 10^4^ cells/well in 6-well cell culture plates. *β*-Galactosidase (*β*-Gal) staining was performed using the Senescence Detection Kit (Millipore) according to the manufacturer's instructions. In brief, SMCs were fixed with the 4% paraformaldehyde and incubated with a staining mix at 37°C. *β*-Gal-positive cells were identified by intense blue color in the nucleus, and they were counted manually in triplicate images. The average from three images was used to calculate the percentage of *β*-Gal-positive cells. *β*-Galactosidase activity was detected using a Mammalian *β*-galactosidase Assay Kit (Thermal Scientific) according to the manufacturer's instructions. In brief, 50 *μ*L of cell extract was transferred into a microplate well, *β*-galactosidase assay reagent (50 *μ*L) was added into the well, the mix was incubated at 37°C for 30 min, and absorbance was read at 405 nm in Cytation 5 microplate reader (Bio-Tek).

### 2.3. Autophagy and Mitophagy Assay

Autophagy was quantified using an Autophagy Assay Kit (Abcam) according to the manufacturer's instructions. The kit measures autophagic vacuoles in live cells using a dye with spectral characteristics similar to FITC that selectively labels autophagic vacuoles. Autophagosome number was quantified by a manual count of FITC-positive dots in 5 viewing fields (each field contains 10–15 cells) together with cell number per field (DAPI). The average autophagic vacuole number per cell is shown in figures. Mitophagy Detection Kit (Dojindo Molecular Technologies, Japan) [16] was used to quantify mitophagy according to the manufacturer's instructions. In brief, the kit is composed of Mtphagy dye (reagent for detection of mitophagy) and Lyso dye (reagent for detection of lysosomes), and when mitophagy is induced, the damaged mitochondria fuse to the lysosome and the Mtphagy dye emits a high fluorescence. The cells were incubated with dye mixture and imaged with Leica TCS SP8 MP inverted spectral confocal microscope (Mtphagy Dye, Ex: 488 nm/Em: 650; Lyso dye, Ex:488/Em:594 nm).

### 2.4. Western Blot Assay

Equal amounts of crude cell lysate or purified mitochondrial fraction (20 *μ*g) were separated by SDS-PAGE and transferred onto PVDF membranes (Millipore). The primary antibodies for LC3B (cat# L7543) and *β*-actin (cat# A3854) were from Sigma and for TOMM20 (cat# ab56783), P16 (CDKN2A/p16INK4a, cat# ab54210), P21 (cat# ab188224), and PINK1 (cat# ab23707) from Abcam. Nrf2 antibody was from Novus Biologicals (cat# NBP1-32822). Sirt3 antibody (cat# #5490) was from cell signaling. Immunoblot imaging and densitometry were performed with ChemiDoc gel documentation system using Gel imaging software (Bio-Rad). To detect the protein expression in mitochondria, the mitochondrial fraction was isolated using a Mitochondria Isolation Kit (Thermo Fisher) according to the manufacturer's instructions. TOMM20 was used as a mitochondrial marker.

### 2.5. SiRNA Transfection

Transfection of SMCs with siRNA was performed using GeneMute reagent (SignGene Lab) according to the manufacturer's instructions. Aliquots of 10^4^ cells were plated on 6-well plates on the day before transfection and grown to 70% confluence. The cells were then transfected with 10 *μ*M PINK1 siRNA, Nrf2 siRNA, Sirt3 siRNA, or control (scrambled) siRNA (Santa Cruz Biotechnology Inc) + 100 *μ*L GeneMute reagent for 6 h. Following an incubation period of 48 h, the protein levels were measured by immunoblotting.

### 2.6. Mitochondrial DNA Damage

Mitochondrial DNA (mtDNA) damage was quantified using mouse RT-PCR Mitochondrial DNA Damage Analysis Kit (Detroit R&D) according to the manufacturer's instructions. The kit quantifies damaged 8.8 kb mtDNA sequences by measurement of the replicated DNA with real-time PCR following QPCR analysis. The 25 *μ*l PCR amplification reaction mixtures contained 12.5 *μ*l of 2xSYBR Premix Taq, 1.0 *μ*l of each primer (10 *μ*M), 0.25 *μ*l of 50xROX II reference dye, 9.25 *μ*l of ddH_2_O, and 1.0 *μ*l of DNA. Real-time PCR conditions were initial denaturation at 95°C for 20 s, followed by 35 cycles of denaturation at 95°C for 3 s and annealing/extension at 58°C for 30 s. The postamplification melting curve analysis was performed to confirm whether the nonspecific amplification was generated. All samples including standard were amplified in duplicate. The standard curve was generated by plotting standard Ct values vs. standard concentration, and mtDNA level in sample was calculated from the standard curve. The *R*^2^ for each standard curve was ≥0.99. In each run, negative and positive controls and a standard curve were included.

### 2.7. Mitochondrial Membrane Potential (MMP) Assay

MMP was quantified in live cells using TMRE Mitochondrial Membrane Potential Assay Kit (Abcam) according to the manufacturer's instructions. In brief, cells were seeded on a 96-well plate (15 × 10^3^ cells/well), stained with fluorescent dye tetramethyl rhodamine ester (TMRE) for 30 min, and the fluorescence signal was immediately measured in a microplate reader (excitation/emission: 549/575 nm).

### 2.8. Mitochondrial DNA (mtDNA) Copy Number

The mitochondrial DNA copy number was determined using a Mouse Mitochondrial DNA Copy Number Assay Kit (Detroit R&D) according to the manufacturer's instructions. In brief, 5 ng of reference and genomic DNA samples were prepared and mixed with primer solution, QPCR master mix, and ddH_2_O to make a 20 *μ*l volume. The PCR condition was as follows: 95°C for 20 s, denaturation; 52°C for 20 s, annealing; 72°C for 45 s, extension. The difference (∆∆Cq) between ∆Cq (mtDNA) and ∆Cq (single copy reference) was calculated. The mtDNA copy number of the target sample was calculated as reference sample mtDNA copy number × 2∆∆Cq.

### 2.9. Cytochrome C Oxidase Assay

Cytochrome C oxidase activity was measured with Cytochrome C Oxidase Assay Kit (Millipore-Sigma) in accordance with manufacturer's instructions.

### 2.10. Statistics

All numeric data are expressed as mean ± SEM. Statistical analysis was performed with GraphPad Prism 7.0. Unless otherwise specified, one-way nonparametric ANOVA tests were performed to determine statistical significance. All experiments were repeated at least 3 times. Differences were considered significant at *P* < 0.05.

## 3. Results

### 3.1. IGF-1 Reversed the Aging Phenotype of Long-Term Cultured Aortic SMC

**Figure 1:**
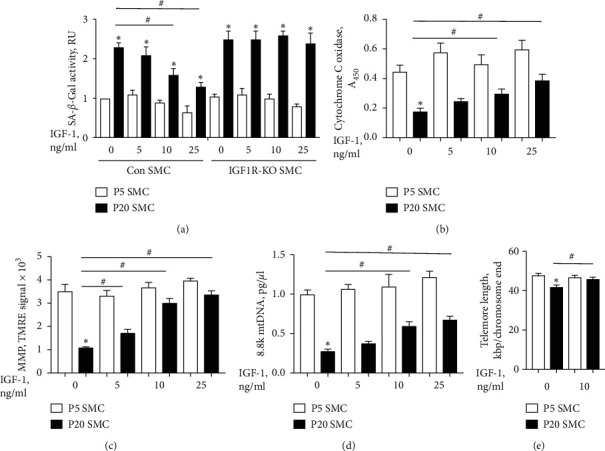
IGF-1 reversed aging phenotype of long-term cultured vascular SMC. Aortic SMCs were isolated from control mice (Con SMC) and mice with IGF-1 receptor deficiency (IGF-1R-KO SMC) and cultured until passage 5 (P5) or passage 20 (P20). Cells were exposed to IGF-1 for 12 h, and activity of senescence-associated *β*-galactosidase (SA-*β*-Gal) (a), cytochrome C oxidase (b), mitochondrial membrane potential (MMP) (c), mitochondrial DNA damage (d), and DNA telomere length (e) was quantified using commercial assays in accordance with manufacturer's instructions. ^*∗*^*P* < 0.05 for P20 vs. P5 SMC. ^#^*P* < 0.05 for IGF-1-treated P20 SMC vs. untreated P20 SMC. Empty bars, P5 SMC; solid bars, P20 SMC.

Prolonged cultivation of primary cells promotes multiple features of cellular aging, including induction of cell senescence and mitochondrial dysfunction. Thus, long-term cultured primary cells are considered a model of cellular aging [17]. To investigate a potential effect of IGF-1 on cellular aging, we performed experiments with cultured short-term (passage 5, P5) and long-term (passage 20, P20) aortic SMC isolated from control mice (Con SMC) and from mice with IGF-1 receptor deficiency (IGF-1R-KO SMC). We quantified cell senescence, indices of mitochondrial dysfunction and DNA telomere length in control SMC at P20 vs. P5. The activity of senescence-associated *β*-galactosidase (SA-*β*-Gal) was upregulated by 2.2-fold in P20 compared to P5 cells (Figure 1(a)). Consistent with these results, we found that a significantly larger number of P20 cells were positive for *β*-Gal compared to P5 cells (P20: 38 ± 4%, P5: 15 ± 3%, SA-*β*-Gal + cells) (Suppl. Figure 1).

Senescence-associated mitochondrial dysfunction is a hallmark of cellular aging [1] and is characterized by reduced oxidative phosphorylation, decreased mitochondrial membrane potential (MMP), and increased mitochondrial DNA (mtDNA) damage [18]. P20 SMC had a 2.5-fold reduction in activity of cytochrome C oxidase and a 3.2-fold decrease in MMP, suggesting mitochondrial dysfunction (Figure 1). P20 cells had marked decrease in amplification of 8.8k mtDNA sequence compared to P5 cells (Figure 1(d)), indicating elevated mtDNA damage. Telomeres are the protective caps on the ends of DNA strands. The length of telomeres in somatic cells decreases with age and may predict lifespan [19]. P20 SMC had a significant reduction in telomere length compared to P5 cells (Figure 1(e)).

Treatment with IGF-1 for 12 hrs induced no significant changes in SA-*β*-Gal activity and *β*-Gal staining in P5 cells. However, IGF-1 dose dependently (5–25 ng/ml) reduced SA-*β*-Gal activity and *β*-Gal staining in P20 Con cells, and these effects were not observed in IGF-1 receptor-deficient P20 SMCs (Figure 1(a) and Suppl. Figure 1) indicating that IGF-1 reduced cell senescence via an IGF-1 receptor-dependent mechanism. Short-term treatment with IGF-1 dose dependently increased cytochrome C oxidase activity and MMP levels and prevented mtDNA damage in P20 cells. We used IGF-1 in the minimum effective dose (i.e., 10 ng/ml) throughout the study. IGF-1 (10 ng/ml) prevented a reduction in telomere length in P20 SMC (Figure 1(e)). P5 and P20 SMCs were treated with IGF-1 (10 ng/ml) for 24 and 72 hrs to test long-term IGF-1's effect (Suppl. Figure 2). Long-term treatment with IGF-1 induced no changes in SA-*β*-Gal activity and telomere length in P5 cells. However, IGF-1 significantly decreased SA-*β*-Gal activity and prevented reduction in telomere length in P20 cells after 24 and 72 hours of treatment (Suppl. Figure 2). Taken together, these results indicate that IGF-1 reversed the cellular aging phenotype.

### 3.2. IGF-1 Increased Autophagy and Mitophagy in SMC

Autophagy is an in-bulk degradative pathway that turns over redundant or damaged organelles and protein aggregates [20], and mitophagy is the specific removal of dysfunctional mitochondria through autophagy [5]. Decreased autophagy and mitophagy with age has been reported extensively in a variety of systems [5, 21, 22], suggesting that autophagy upregulation may counteract aging. We hypothesized that IGF-1's ability to reduce cellular aging is mediated by enhanced autophagy. The serum contains significant IGF-1 levels [23], and therefore, we cultured cells with/without IGF-1 in a serum-starved media. Since serum starvation is a potent autophagy/mitophagy inducer [24, 25], IGF-1 effects were tested in cells with basal enhancement in autophagy. This approach increased sensitivity of autophagy detection in experiments with “aged” cells with relatively low autophagy/mitophagy levels.

**Figure 2:**
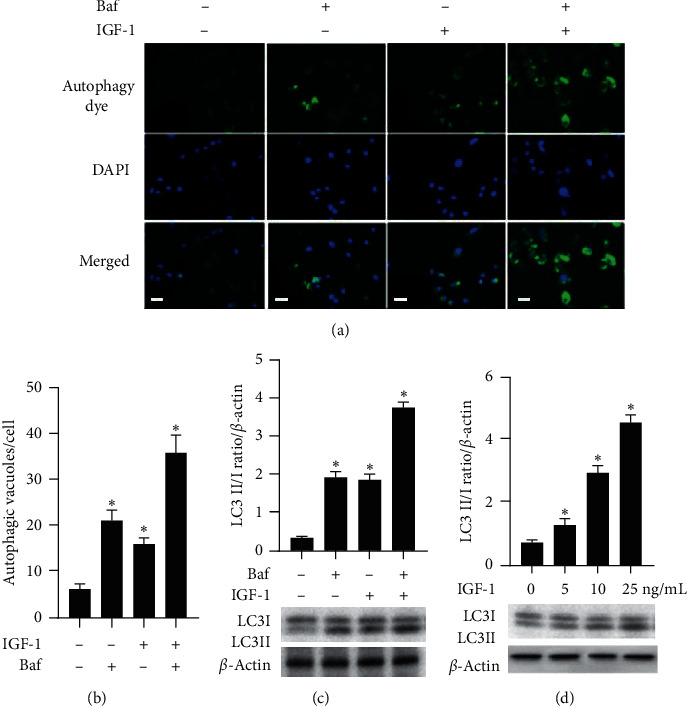
IGF-1 upregulated autophagy in P20 SMC. P20 SMCs were treated with 10 ng/ml IGF-1, with 10 *μ*M Bafilomycin (Baf), or with combination of IGF-1 and Baf and stained with dye labelling autophagic vacuoles and counterstained with DAPI (a, b). Autophagic dye signal was localized in spherical vacuoles in the perinuclear region of the cell and in foci distributed throughout the cytoplasm. Data were shown as a total number of autophagic vacuoles per cell. LC3 II/I ratio was quantified by immunoblotting/densitometry (c, d), and the results were normalized per *β*-actin levels. ^*∗*^*P* < 0.05 for IGF-1-treated vs. untreated cells. Scale bar, 100 *μ*m.

Microtubule-associated protein light chain 3 (LC3) is a well-established marker used to monitor autophagy by measuring conversion of LC3 I to the faster migrating form LC3 II hereafter referred to as LC3 II/I ratio or LC3B conversion [26]. Treatment with IGF-1 for 12 hrs dose dependently increased LC3 II/I ratio in aged P20 cells, indicating that IGF-1 upregulated autophagy (Figure 2). To quantify constitutive autophagosomes in P20 cells, we used a dye that selectively labels autophagic vacuoles. IGF-1 (10 ng/ml) markedly increased the number of autophagic vacuoles (autophagosomes) per cell (Figure 2(a)). Bafilomycin A1 (Baf) is a V-ATPase inhibitor that inhibits autophagosome-lysosome fusion and stops autophagosome turnover causing LC3 II accumulation [27]. Baf evoked a potent upregulation of autophagy in SMC and cell cotreatment with IGF-1 (12 hrs), and Baf induced an additional increase in autophagosomes (Figure 2). Treatment with IGF-1 for 72 hrs also significantly increased LC3 II/I ratio in P20 cells and cotreatment with Baf-induced additional elevation of LC3 conversion (Suppl. Figure 3). Baf blocks autophagic flux by preventing autophagosome digestion. Thus, the observed increase in autophagic vacuoles and LC3 II in Baf-treated cells is due to prevention of the vacuole digestion by the lysosome. These results suggest that IGF-1 upregulated the number of autophagic vacuoles by stimulating autophagosome formation, rather than by inhibiting degradation of autophagosomes. Treatment with Baf also increased LC3 II/I ratio, and IGF-1 induced a further increase in the presence of Baf (Figure 2(c) and Suppl. Figure 3). These data show that IGF-1 promotes autophagy in P20 cells potentially through increase in autophagosome formation.

**Figure 3:**
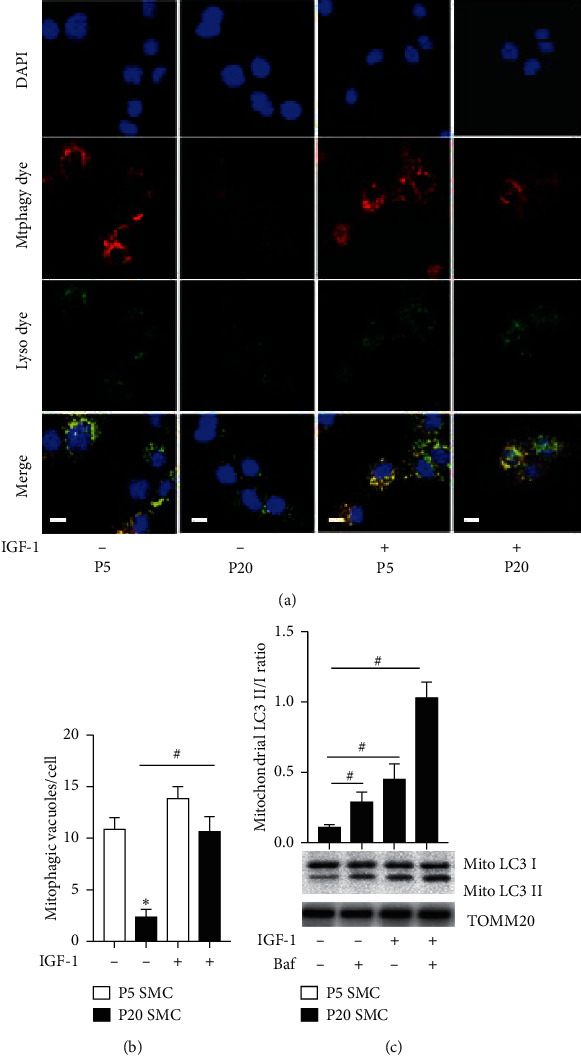
IGF-1 upregulated mitophagy. P5 and P20 SMC were exposed to 10 ng/ml IGF-1 for 12 h and stained with Mtphagy and Lyso dyes using the Mitophagy Detection Kit (a, b). Mtphagy dye emits a high fluorescence when mitochondria fuse with lysosome, and this signal was detected by 594 nm filter; Lyso dye signal was assessed by 488 nm filter. Overlapping signal of Mtphagy and Lyso dyes (merge) was localized exclusively in the perinuclear region of a cell. A number of vacuoles double-positive for Mtphagy and Lyso was quantified per cell and shown in the graph. P20 cells were treated with IGF-1, or Baf, or with combination of IGF-1 + Baf; mitochondrial fraction was isolated, and mitochondrial LC3 II/I ratio was quantified by immunoblotting/densitometry (c). ^*∗*^*P* < 0.05 for untreated P20 vs. untreated P5 SMC. ^#^*P* < 0.05 for cells treated with IGF-1 or Baf or combination of IGF-1 and Baf vs. untreated SMC. Empty bars, P5 SMC; solid bars, P20 SMC. Scale bar, 100 *μ*m.

IGF-1 increased mitochondrial biogenesis in cancer cell lines [10]. To evaluate IGF-1's effect on mitochondrial abundance in vascular SMC, we quantified mitochondrial mass and mitochondrial DNA copy number in P20 cells (Suppl. Figure 4). We found that IGF-1 increased mitochondrial mass and mtDNA copies in P20 SMC. To quantify the potential effect of IGF-1 on mitophagy, we stained live cells with a dye (Mtphagy dye) that becomes fluorescent when damaged mitochondria fuse with lysosomes [16]. The dye signal was dramatically reduced in P20 compared to P5 cells (Figure 3(b)), showing that inhibition of mitophagy was associated with the aging phenotype acquired by P20 cells. IGF-1 (10 ng/ml for 12 hrs) significantly increased Mtphagy dye signal in P20 cells: in fact, dye fluorescence in IGF-1-treated P20 cells was similar to the one detected in untreated P5 cells. Long-term P20 cells exposure to IGF-1 (10 ng/ml for 72 hrs) also significantly increased Mtphagy dye signal (Suppl. Figure 5). We evaluated IGF-1's effect on LC3B conversion in the mitochondrial fraction of P20 cells by quantification of LC3 II/I ratio. Mitochondrial LC3B conversion was increased by IGF-1, and we observed an additional increase in LC3B conversion when cells were cotreated with IGF-1 and Baf (Figure 3(c)).

### 3.3. IGF-1 Downregulated Cyclin-Dependent Kinase Inhibitors p16 and p21

**Figure 4:**
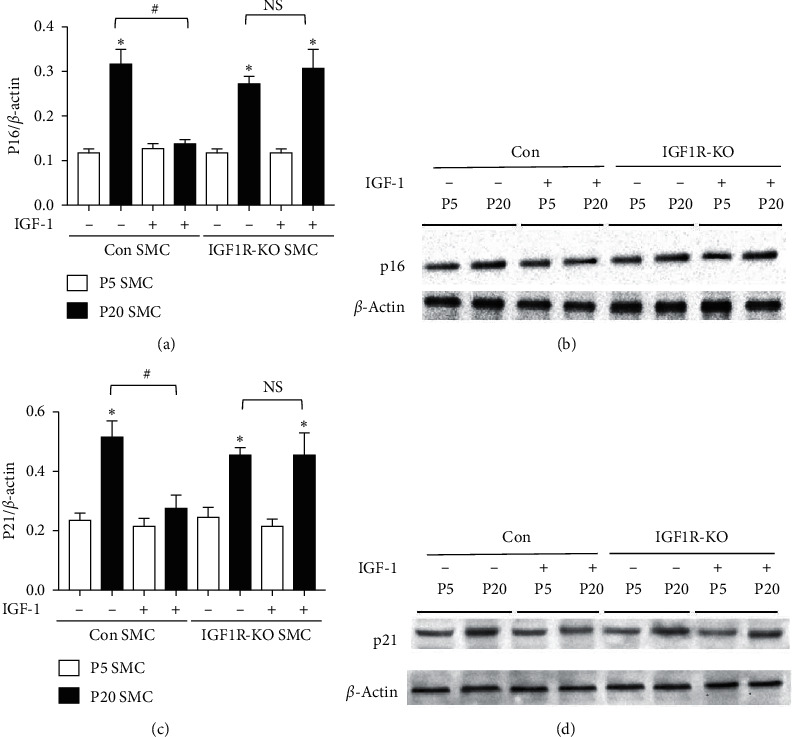
IGF-1 downregulated cyclic-dependent kinase inhibitors p16 and p21. P5 and P20 Con SMC and IGF-1R-KO SMC were exposed to 10 ng/ml IGF-1 for 12 hours, and p16 and *β*-actin levels (a, b) or p21 and *β*-actin levels (c, d) were quantified by immunoblotting. ^*∗*^*P* < 0.05 for P20 SMC vs. P5 SMC. ^#^*P* < 0.05 vs. no IGF-1.

The transition through the G_1_ phase and entry into the S-phase of the cell cycle requires activation of the cyclin/cyclin-dependent kinase (CDK) complex, whose kinase activity is inhibited by CDK inhibitors, including p16 and p21 [28]. Upregulation of p16 and p21 is a primary mechanism arresting cell proliferation and inducing cell senescence [29]. Elevated p16 and p21 levels were found in aged human skin [30] and animal aging models [31] and were reported for several *in vitro* aging models [32]. Similarly, aged P20 SMC had upregulated levels of p16 and p21 compared to P5 cells consistent with cellular aging (Figure 4). P20 Con cell treatment with IGF-1 reduced CDK inhibitors levels: in fact, both p16 and p20 expression levels in IGF-1-treated P20 cells were not different from nontreated P5 cells (Figure 4). IGF-1 did not change p16 and p21 levels in IGF-1R-KO SMC, showing that IGF-1-induced reduction in CDK inhibitors was mediated via an IGF-1 receptor-mediated signaling pathway.

### 3.4. IGF-1 Upregulated Mitophagy via the Nrf2/Sirt3 Pathway

Mitochondrial homeostasis is maintained by a balance between mitophagy-dependent clearance of damaged mitochondria and generation of functional mitochondria through stimulation of mitochondrial biogenesis. Nuclear respiratory factor 2 (Nrf2) and sirtuins have been implicated in the coordination of mitophagy and mitochondrial biogenesis. Nrf2 is a transcription factor that controls cell cycle progression as well as mitochondrial biogenesis [33]. Importantly, a synthetic 1,4-diphenyl-1,2,3-triazole compound, originally designed as an Nrf2 activator, was found to promote mitophagy [34]. Sirtuin-3 (Sirt3) is a mitochondrial deacetylase and a regulator of mitochondrial biogenesis and mitophagy [35], and Sirt3 expression declines with aging [36].

**Figure 5:**
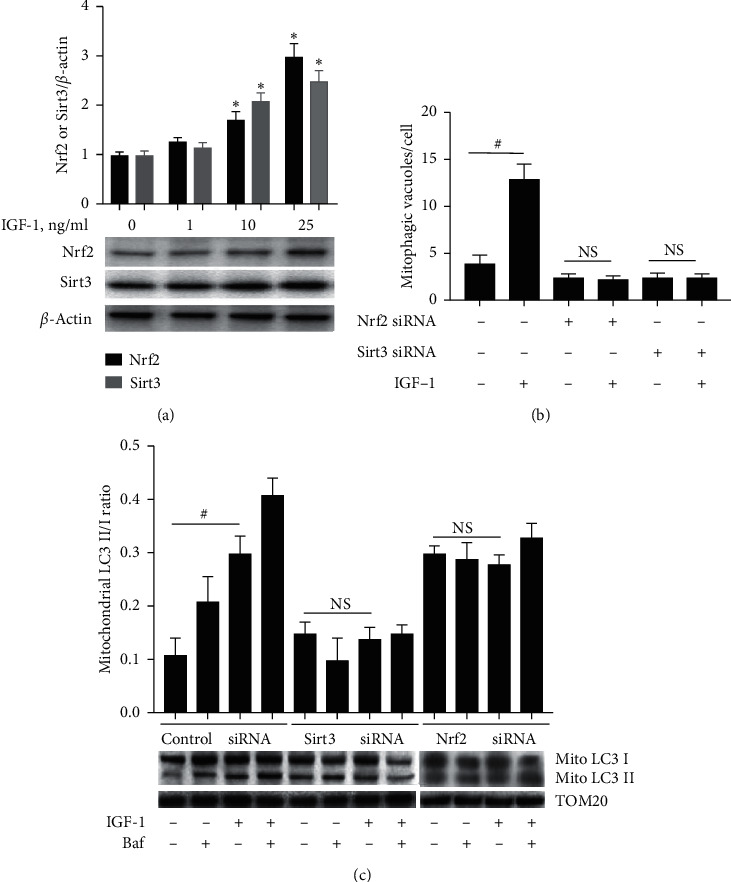
IGF-1 dose dependently upregulated Nrf2 and Sirt3 expression (a) and Nrf2/Sirt3 pathway mediated IGF-1-induced mitophagy activation (b, c). A, P20 SMC was treated with IGF-1 and Nrf2, and Sirt3 and *β*-actin levels were quantified by immunoblotting. (b, c) P20 SMC was transfected with 5 nM Nrf2 siRNA or Sirt3 siRNA or scrambled siRNA (control), and after 48 hours, cells were exposed to IGF-1, bafilomycin (Baf), or combination of IGF-1 + Baf. Mitophagy was detected by live cell staining with Mtphagy + Lyso dyes and quantifying a number of mitophagic vacuoles/cell (b) or by assessing mitochondrial LC3 II/I ratio (c). ^*∗*^*P* < 0.05 vs. untreated cells; ^#^*P* < 0.05 for SMC with IGF-1 vs. no IGF-1.

We hypothesized that IGF-1 increased Nrf2 and/or Sirt3 levels and that this mechanism mediated the IGF-1's antiaging effect. IGF-1 dose dependently increased both Nrf2 and Sirt3 protein expression in P20 cells (Figure 5(a)). To demonstrate involvement of the Nrf2/Sirt3 axis in IGF-1-induced effects, expression of Nrf2 or Sirt3 was downregulated by using specific siRNA. Nrf2-specific siRNA decreased Nrf2 protein by 59 ± 7% and Sirt3-specific siRNA downregulated Sirt3 by 77 ± 10% (*P* < 0.05 compared to control, scrambled siRNA, Suppl. Figure 6). IGF-1 increased the number of mitophagic vacuoles and mitochondrial LC3 II/I ratio in control siRNA-transfected cells (Figures 5(b) and 5(c)). However, both IGF-1-induced increase in mitophagic vacuoles and mitochondrial LC3 II/I ratio were completely blocked either by Nrf2 or Sirt3 siRNA, demonstrating that a functional Nrf2/Sirt3 axis is required for IGF-1-induced activation of mitophagy.

### 3.5. Inhibition of Mitophagy Blocked IGF-1-Induced Antiaging Effects

**Figure 6:**
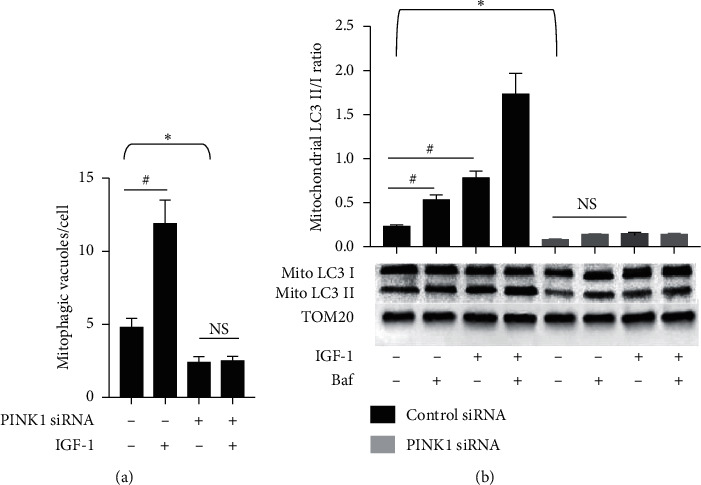
PINK1 silencing inhibited mitophagy in SMC. P20 SMCs were transfected with 5 nM PINK1 siRNA or scrambled siRNA (control), and after 48 hours, cells were exposed to IGF-1, bafilomycin (Baf), or combination of IGF-1 + Baf. Mitophagy was determined by quantifying of number of mitophagic vacuoles/cell (a) or by assessing of mitochondrial LC3 II/I ratio (b). Black bars, control siRNA-transfected cells; grey bars, PINK1 siRNA-transfected cells. ^*∗*^*P* < 0.05 PINK1 siRNA vs. control siRNA; ^#^*P* < 0.05 for SMC with IGF-1 vs. no IGF-1.

**Figure 7:**
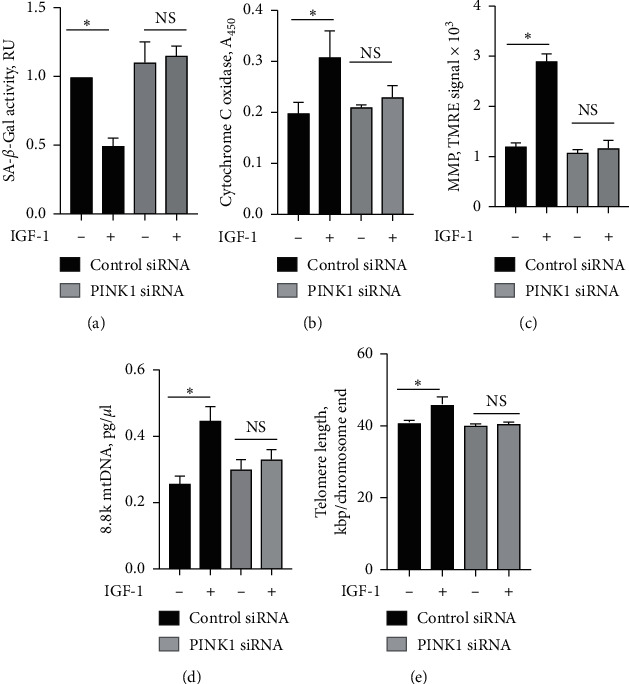
PINK1 silencing blocked IGF-1-induced effect on cellular aging. P20 SMC was transfected with 5 nM PINK1 siRNA (black bars) or scrambled siRNA (control, grey bars), and after 48 hours, cells were exposed to IGF-1. Activity of senescence-associated *β*-galactosidase (SA-*β*-gal) (a), cytochrome C oxidase (b), mitochondrial membrane potential (MMP) (c), mitochondrial DNA damage (d), and DNA telomere length (e) was quantified using commercial assays. ^*∗*^*P* < 0.05 for SMC + IGF-1 vs. no IGF-1.

During mitophagy, PTEN-induced putative kinase 1 (PINK1) accumulates on the surface of dysfunctional mitochondria to facilitate removal of toxic mitochondrial products [37]. PINK1 downregulation prevents colocalization of mitochondria and lysosomes, suggesting inhibition of mitophagosome formation [38], and siRNA-induced PINK1 knockdown also significantly decreases the LC3 II/I ratio in cardiomyocytes [39]. Taken together, these results indicate that PINK1 silencing inhibits mitophagy. We performed PINK1 silencing with PINK1-specific siRNA (control: scrambled siRNA) to suppress mitophagy in P20 cells. PINK1 siRNA reduced PINK1 protein levels in P20 SMC by >60% compared to control (Suppl. Figure 6). PINK1 silencing decreased mitophagy by 57 ± 7% and 60 ± 6% compared to control as was assessed by quantification of mitophagic vacuoles and measurement of mitochondrial LC3 II/I ratio, respectively (Figure 6). IGF-1 activated mitophagy in control cells; however, this effect was completely abolished by PINK1 silencing (Figure 6). To test whether activation of mitophagy mediates IGF-1-induced antiaging effect, we quantified cell senescence, indices of mitochondrial dysfunction (cytochrome C oxidase activity, MMP, and mtDNA damage), and DNA telomere length in P20 cells with PINK1 silencing. IGF-1 significantly reduced SA-*β*-Gal activity, increased MMP, activated cytochrome C oxidase, and increased DNA telomeres in P20 cells transfected with control siRNA, and all these effects were completely blocked by PINK1 silencing (Figure 7) indicating that IGF-1-induced antiaging effect is dependent on activation of mitophagy.

## 4. Discussion

Here, we report that IGF-1 reversed the aging phenotype of long-term cultured aortic SMC. IGF-1 decreased cell senescence, elevated activity of cytochrome C oxidase, increased MMP, reduced mitochondrial DNA damage, and inhibited shortening of DNA telomeres. These effects were associated with upregulated autophagy and mitophagy, decreased expression of CDK inhibitors p16 and p21, and upregulated levels of Nrf2 and Sirt3. SiRNA-induced inhibition of either Nrf2 or Sirt3 expression completely blocked activation of mitophagy induced by IGF-1, indicating that the Nrf2/Sirt3 pathway was required for activation of mitophagy. We utilized PINK1 silencing to block mitophagy activation in aged cells. We found that PINK1 silencing abolished IGF-1-induced activation of mitophagy and that IGF-1 was not able to reverse the aging phenotype of aged cells with silenced PINK1. Thus, our data indicate that IGF-1 induced an antiaging effect via activation of mitophagy through a Nrf2/Sirt3-dependent mechanism. Importantly, we showed that age-related changes in the cellular level can be at least partially reversed by relatively short-term IGF-1 treatment. Our study suggests a therapeutic potential of IGF-1 to stimulate mitophagy and slow cellular aging.

In the current report, we explored the use of long-term cultured primary cells, as a model of cellular aging. SMC primary culture is a mixture of fast dividing precursors, a majority of differentiated SMC, as well as subpopulation of passage-dependent senescent cells. Thus, the primary cell response to IGF-1 depends on passage number and specific contribution of each cell subpopulation in tested parameters. This is an inherent restriction when observations from low and high passages are compared and is a limitation of using primary cell cultures. We made attempt to characterize specific P5 and P20 cell composition by assessment of number of SA-*β*-Gal + cells and by visualizing cells with activated mitophagy in P5 and P20 cultures with and without IGF-1. P20 cells contain a larger number of SA-*β*-Gal + cells compared to P5 culture and IGF-1 reduced SA-*β*-Gal-positivity of P20 cells (Suppl. Figure 1). The majority of P5 cells were mitophagy active vs. virtually none in P20 culture. Importantly, IGF-1 markedly increased number of mitophagy active cells in P20 SMC (Figure 3). Our working hypothesis is that IGF-1 induces replenishing of P20 cells via mitophagy-mediated clearance of mitochondria and suppressing of cell senescence. This hypothesis is consistent with literature data indicating prosurvival and mitogenic effects of IGF-1 [6, 40, 41].

Cellular aging is a driven force of tissue-specific aging (i.e., vascular aging) and aging of the entire organism [3], and only using *in vitro* models allow testing specific role of complex cellular pathways (such as cell senescence, autophagy, and mitochondrial function) in aging. In addition, availability of genetically modified mice justifies using murine primary cells to identify specific molecular targets linking cellular aging with tissue aging and aging of the entire organism. We demonstrated that murine P20 SMC acquired several features of cellular aging including induction of cell senescence, mitochondrial dysfunction, and telomere shortening consistent with data reported for other models of cellular aging [17]. To our knowledge, this is the first report establishing an *in vitro* aging model with vascular SMC. Dysfunctional SMCs are a hallmark of vascular aging and aging-induced pathologies such as atherosclerosis and aortic aneurism. Therefore, long-term cultured SMC may be helpful to study aging and aging-associated diseases.

Both cell senescence and mitochondrial dysfunction are features of cellular aging [1], and the two have been independently identified as important drivers of aging [42]. Overexpression of CDK inhibitors p16 or p21 induced cell senescence [43], and elevated p16 and p21 levels were associated with aging [30–32], showing that p16 and p21 are involved in development of cell senescence-induced aging phenotype. We report here that IGF-1 downregulated p16 and p21 levels, decreased cell senescence, and reversed the aging phenotype acquired by P20 cells. These results are in line with the critical role of CDK inhibitors in development of senescence-induced aging. Liver-specific knockdown of IGF-1 decreased Nrf2 levels in mouse aortas and was associated with exacerbation of endothelial dysfunction, increased oxidative stress, and apoptosis, mimicking the vascular aging phenotype [44]. This report suggested that activation of IGF-1 signaling may upregulate Nrf2 expression and induce an antiaging effect. Activation of Nrf2 was shown to promote mitophagy and support mitochondrial integrity in hepatic cells [45], indicating the potential role of Nrf2 in mitophagy activation. Our novel finding that IGF-1 upregulated Nrf2 and activated mitophagy in aged vascular SMCs, and that Nrf2 knockdown blocked mitophagy activation induced by IGF-1, establishing Nrf2 as an important mechanistic link between IGF-1 and mitophagy.

**Figure 8:**
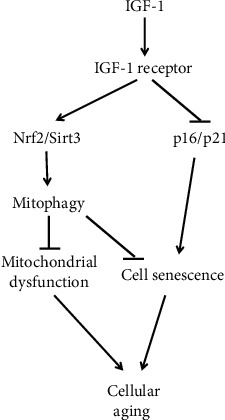
Hypothetical molecular mechanism mediating IGF-1's effect on cellular aging. IGF-1 upregulates Nrf2 and Sirt3 and downregulates CDK inhibitors p16 and p21. Activation of Nrf2/Sirt3 pathway stimulates mitophagy, and p16/p21 downregulation leads to reduction in cell senescence. Both mechanisms (restoring mitochondrial function and prevention of cell senescence) contribute to antiaging effects induced by IGF-1.

Sirt3 deacetylase has been reported to induce mitophagy in cancer cells [46] and to be involved in prevention of aging-associated degeneration of hematopoietic stem cells [36]. Although exercise has been shown to improve the aging-related reduction in IGF-1 and Sirt3 in neuronal cells in sedentary aged rats [47], the potential role of IGF-1 in regulation of Sirt3 expression is unknown. In the current report, we found that IGF-1 upregulated Sirt3 levels in vascular SMC and that Sirt3 was a critical mediator of IGF-1-induced activation of mitophagy. Overall, our study showed that IGF-1 promoted an antiaging effect in SMC through two major mechanisms: inhibition of cell senescence potentially mediated by downregulation of p16 and p21 and Nrf2/Sirt3-dependent activation of mitophagy (Figure 8). Our data indicate that mitophagy activation is the predominant mechanism contributing to IGF-1's antiaging effect, since our demonstration that specific inhibition of mitophagy (by PINK1 silencing) was sufficient to completely block IGF-1's effect on cellular aging.

Unrepaired DNA damage promotes cellular and vascular aging [48], and specific mitochondrial DNA damage is now viewing as a principal mechanism mediating aging [49]. We found that IGF-1 suppressed mtDNA damage in aged SMC. These results taken together with literature reports suggest that IGF-1′ effect on mtDNA may contribute to antiaging effect and show prospective direction for future investigations.

Aging and atherosclerosis share similar underlying biochemical pathways, and vascular dysfunction and aging are independent risk factors for atherosclerosis [4]. It has been reported that either induction of cell senescence or interruption of autophagic signaling in vascular SMC promoted atherosclerosis and induced features of plaque vulnerability in a murine model [50, 51]. We and others [40, 41, 52] have reported that IGF-1 exerts antiatherosclerotic effects in mice that are mediated (at least in part) by IGF-1 receptor-dependent SMC signaling [15]. The results of the current report suggest that the ability of IGF-1 to reduce cell senescence and activate autophagy in vascular SMC could be a novel mechanism(s) potentially contributing to IGF-1's antiatherosclerotic effects. Interestingly, the beneficial effect of IGF-1 on the cellular aging is in contrast to the reports that inhibition of IGF-1 signaling in invertebrates increased longevity [11] and reports of IGF-1 receptor heterozygosity on longevity in mice [12, 13].

In summary, IGF-1 reversed the aging phenotype acquired by long-term cultured aortic SMC. The IGF-1-induced antiaging effect was associated with decreased expression of CDK inhibitors p16 and p21, upregulated levels of Nrf2 and Sirt3, and activation of mitophagy. IGF-1-induced activation of mitophagy was Nrf2/Sirt3 dependent. PINK1 silencing suppressed mitophagy and completely blocked IGF-1-induced antiaging effect, showing that IGF-1's antiaging effect was dependent on activation of mitophagy. Our data establish a novel role of IGF-1 in promoting autophagy and mitophagy in SMC. Activation of IGF-1 signaling could be a novel strategy to upregulate mitophagy, thereby reducing cell senescence, and slow cellular aging.

## Data Availability

Data and materials related to this work are available upon request.

## Abbreviations

IGF-1: Insulin-like growth factor I
SMC: Smooth muscle cells
MMP: Mitochondrial membrane potential
Nrf2: Nuclear respiratory factor 2
Sirt3: Sirtuin-3 mitochondrial NAD-dependent deacetylase
siRNA: Small interference RNA
mtDNA: Mitochondrial DNA.

## Supplementary Materials

Supplementary Materials Supplementary materials include 6 figures with legends. Suppl.Figure 1 shows *β*-Gal positivity in P5 and P20 cells treated with IGF-1. It supports conclusions drawn from main Figure 1. Suppl.Figure 2 shows that long-term treatment with IGF-1 decreased activity of senescence-associated *β*-galactosidase and reversed reduction in DNA telomere length. These data were obtained by cell treatment with IGF-1 for 24 and 72 hrs, and they complement results generated with short-term IGF-1 exposure (12 hrs) (shown in main Figure 1). Suppl.Figure 3 shows that SMC treatment with IGF-1 (72 hrs) upregulated autophagy in P20 SMC. These data complement data for short-term (12 hrs) treatment shown in Figure 2. Suppl.Figure 4 shows quantification of mitochondrial mass and mitochondrial DNA copy number in P20 cells treated with IGF-1 and concludes that IGF-1 increased mitochondrial mass and mtDNA copies in P20 SMC. Suppl.Figure 5 shows that 72 hrs SMC treatment with IGF-1 upregulated mitophagy consistent with findings shown in Figure 3. Suppl.Figure 6 confirms successful Nrf2, Sirt3, and PINK1 silencing using molecule-specific siRNA.
