# Supplementary material for: Insulin-Like Growth Factor I Prevents Cellular Aging via Activation of Mitophagy
Source: J Aging Res. 2020 Aug 1;2020:4939310. doi: 10.1155/2020/4939310 (PMC7416301; doi:10.1155/2020/4939310)
Supplement: Supplementary Materials — include 6 figures with legends. Suppl.Figure 1 shows β-Gal positivity in P5 and P20 cells treated with IGF-1. It supports conclusions drawn from main Figure 1. Suppl.Figure 2 shows that long-term treatment with IGF-1 decreased activity of senescence-associated β-galactosidase and reversed reduction in DNA telomere length. These data were obtained by cell treatment with IGF-1 for 24 and 72 hrs, and they complement results generated with short-term IGF-1 exposure (12 hrs) (shown in main Figure 1). Suppl.Figure 3 shows that SMC treatment with IGF-1 (72 hrs) upregulated autophagy in P20 SMC. These data complement data for short-term (12 hrs) treatment shown in Figure 2. Suppl.Figure 4 shows quantification of mitochondrial mass and mitochondrial DNA copy number in P20 cells treated with IGF-1 and concludes that IGF-1 increased mitochondrial mass and mtDNA copies in P20 SMC. Suppl.Figure 5 shows that 72 hrs SMC treatment with IGF-1 upregulated mitophagy consistent with findings shown in Figure 3. Suppl.Figure 6 confirms successful Nrf2, Sirt3, and PINK1 silencing using molecule-specific siRNA. [file 4939310.f1.pdf]

SUPPLEMENTS

Supplemental Figure 1. IGF-1 reduced SMC senescence via IGF-1 receptor-dependent mechanism. Aortic SMC were isolated from control mice (Con SMC) and from mice with IGF-1 receptor deficiency (IGF-1R-KO SMC) and cultured until passage 5 (P5) or passage 20 (P20). Cells were exposed to IGF-1 for 12h and β-Galactosidase (β-gal) staining was performed using the Senescence Detection Kit. A, representative images of β-Gal–positive cells. B, quantitative data. *$P<0.05$ for untreated P20 vs. untreated P5 SMC. #$P<0.05$ for P20 Con SMC+IGF-1 vs, untreated P20 Con SMC. Empty bars, P5 SMC, solid bars, P20 SMC.

Supplemental Figure 2. Long-term treatment with IGF-1 decreased activity of senescence-associated β-galactosidase (A) and reversed reduction in DNA telomere length (B). Murine aortic SMC were cultured until passage 5 (P5) or passage 20 (P20). Cells were exposed to IGF-1 for 24 and 72hrs and activity of senescence-associated β-galactosidase (SA β-gal) (A), and DNA telomere length (B) were quantified using commercial assays in according with manufacturer's instructions. *$P<0.05$ for P20 vs. P5 SMC. #$P<0.05$ for IGF-1-treated P20 SMC vs. untreated P20 SMC. Empty bars, P5 SMC, solid bars, P20 SMC.

Supplemental Figure 3. SMC treatment with IGF-1 (72hrs) upregulated autophagy in P20 SMC. P20 SMC were treated with 10 ng/ml IGF-1, or with 10 uM Bafilomycin (Baf), or with combination of IGF-1 and Baf. LC3 II/I ratio was quantified by immunoblotting/densitometry and results were normalized per GAPDH levels. *$P<0.05$ vs. untreated cells. #$P<0.05$ IGF-1+Baf, vs IGF-1.

Supplemental Figure 4. IGF-1 increased mitochondrial mass. A, P20 SMC were stained with MitoTracker Deep Red FM dye and the signal was quantified by confocal microscopy. B, mitochondrial copy number was assessed in P20 SMC by using Mouse mitochondrial DNA copy number kit. *$P<0.05$ for SMC+IGF-1 vs. untreated SMC.

Supplemental Figure 5. SMC treatment with IGF-1 (72hrs) upregulated mitophagy. P5 and P20 SMC were exposed to 10 ng/ml IGF-1 for 72hrs and stained with Mtphagy and Lyso dyes using Mitophagy detection kit (A). The number of vacuoles double positive for Mtphagy and Lyso was quantified per cell and shown on the graph (B). *$P<0.05$ for

untreated P20 vs. untreated P5 SMC. #P<0.05 for IGF-1 vs. no IGF-1. Empty bars, P5 SMC, solid bars, P20 SMC.

Supplemental Figure 6. SiRNA-induced downregulation of Nrf2, Sirt3 and PINK1. P20 SMC were transfected with 5nM Nrf2 siRNA (A) or Sirt3 siRNA (B) or PINK1 siRNA (C) or scrambled siRNA (control) and after 48 hours cells protein expression was assessed by immunoblotting.

Suppl.Fig.1

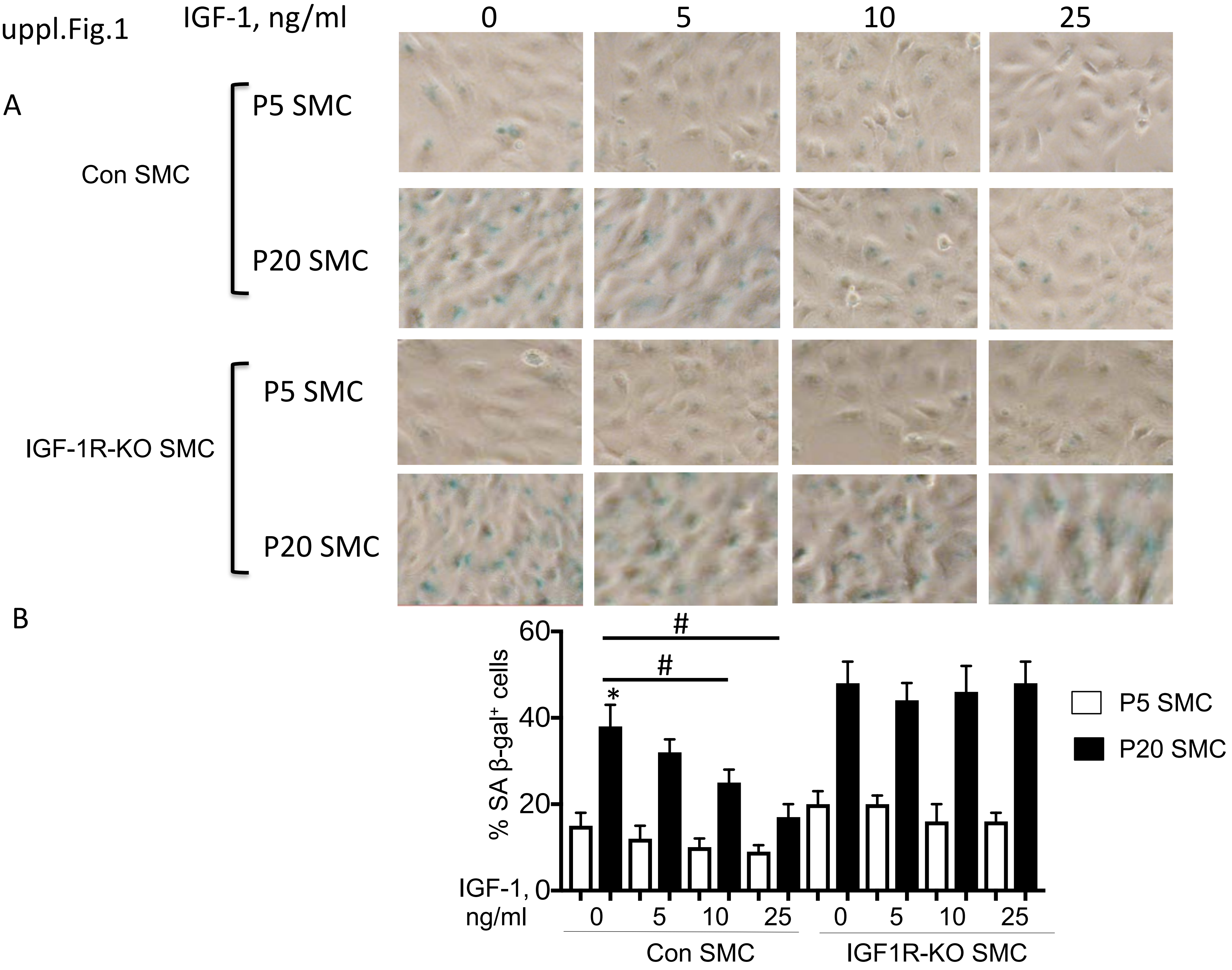

IGF-1, ng/ml
0
5
10
25
A
Con SMC
P5 SMC
P20 SMC
IGF-1R-KO SMC
P5 SMC
P20 SMC
B
% SA β-gal⁺ cells
60
40
20
0
#
#
*
IGF-1, ng/ml
0
5
10
25
0
5
10
25
Con SMC
IGF1R-KO SMC
P5 SMC
P20 SMC

Suppl.Fig.2

A

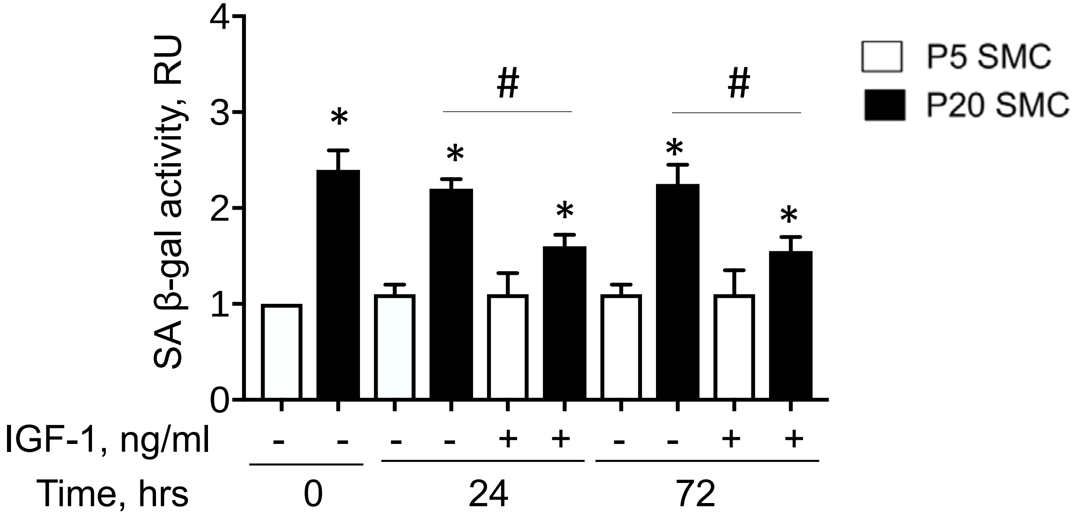

B

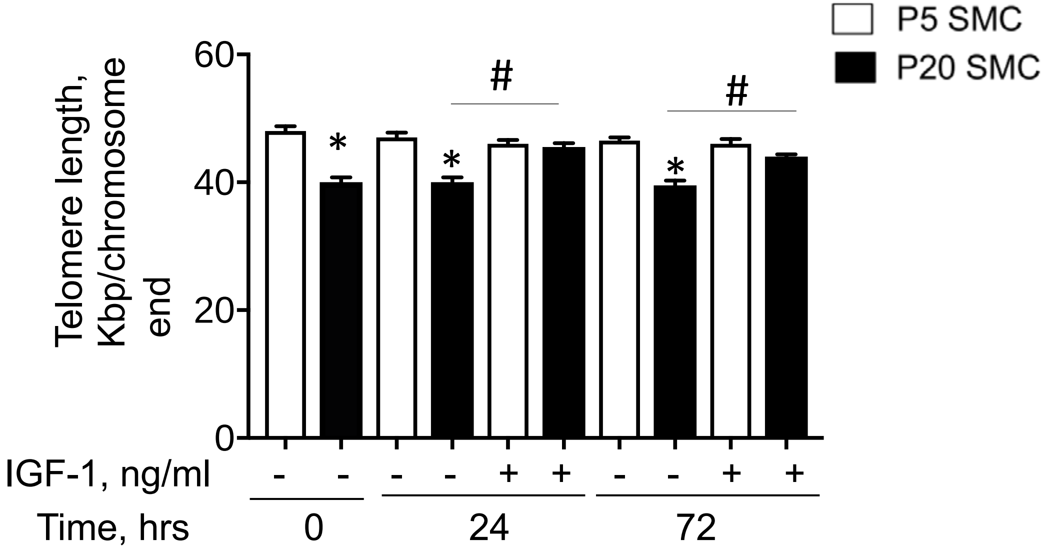

Suppl.Fig.3

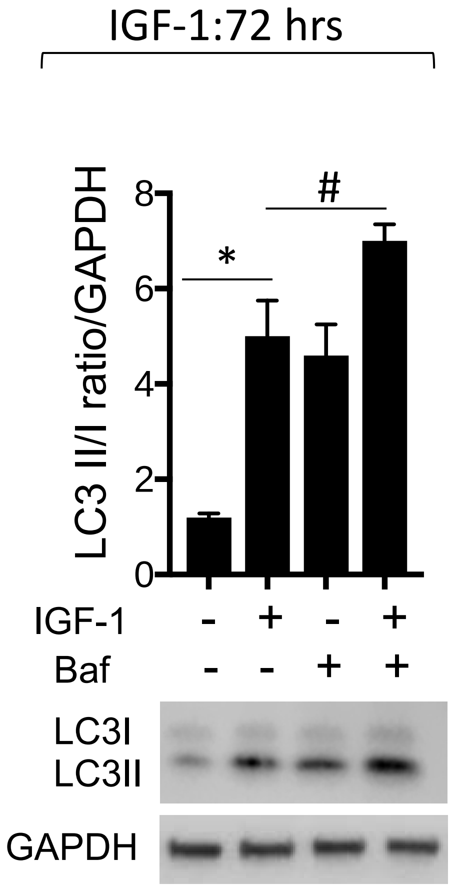
IGF-1:72 hrs
LC3 II/I ratio/GAPDH
8
6
4
2
0
*
#
IGF-1 - + - +
Baf - - + +
LC3I
LC3II
GAPDH

Suppl. Fig.4

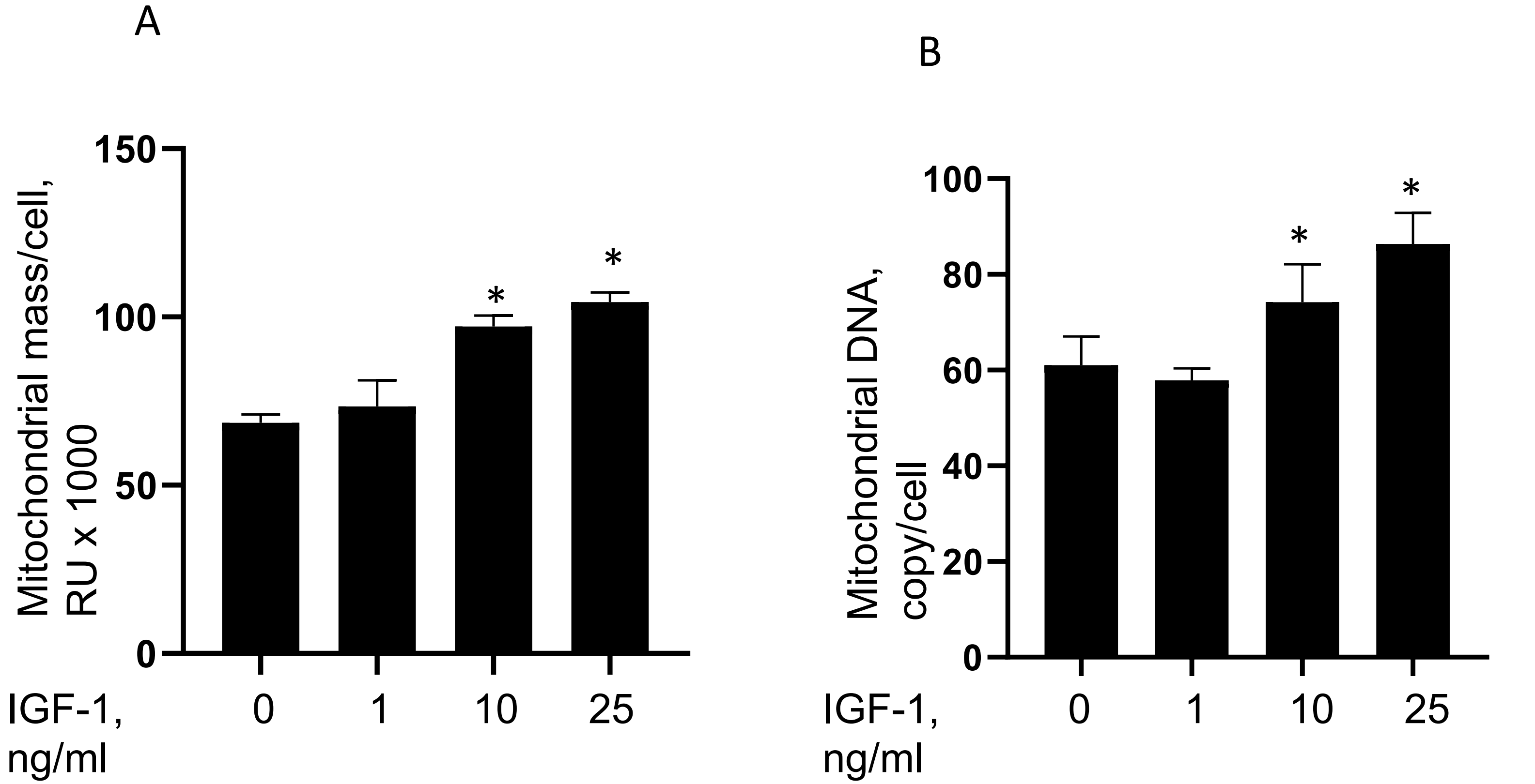
A
150
100
50
0
Mitochondrial mass/cell, RU x 1000
*
*
IGF-1, ng/ml
0
1
10
25
B
100
80
60
40
20
0
Mitochondrial DNA, copy/cell
*
*
IGF-1, ng/ml
0
1
10
25

Suppl.Fig.5

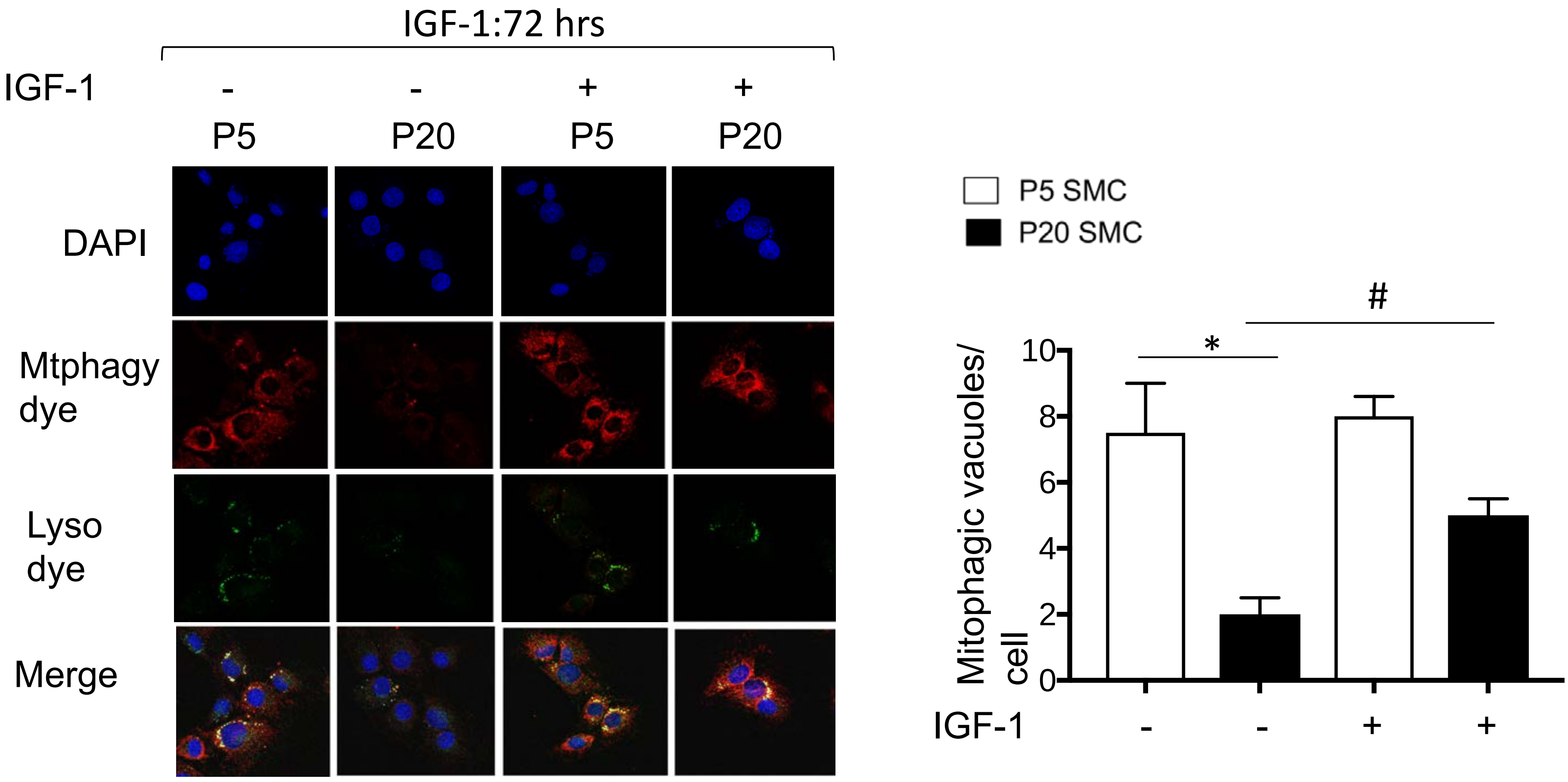
IGF-1:72 hrs
IGF-1
-
-
+
+
P5
P20
P5
P20
DAPI
Mtphagy dye
Lyso dye
Merge
P5 SMC
P20 SMC
Mitophagic vacuoles/ cell
10
8
6
4
2
0
*
#
IGF-1
-
-
+
+

Suppl.Fig.6

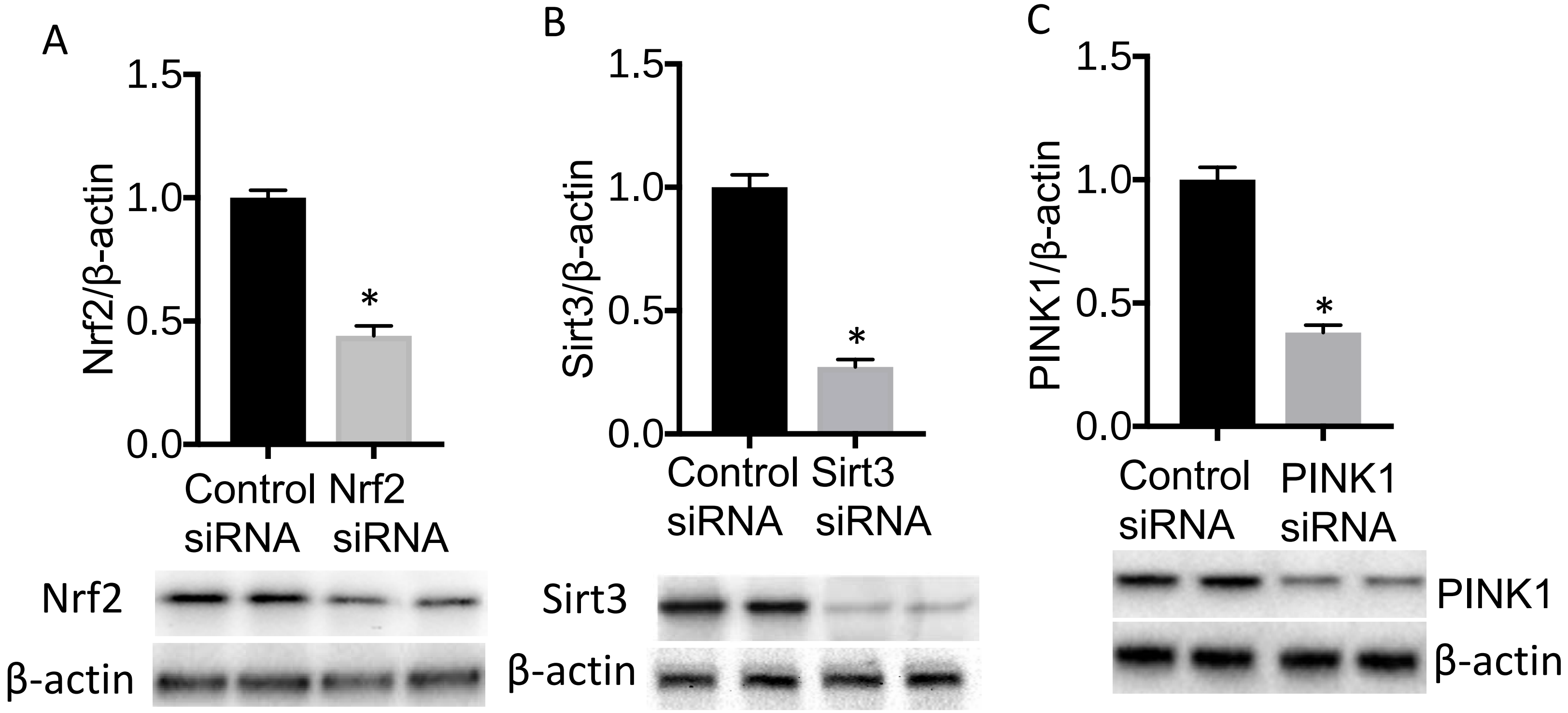
A
1.5
1.0
0.5
0.0
Nrf2/β-actin
*
Control siRNA
Nrf2 siRNA
Nrf2
β-actin
B
1.5
1.0
0.5
0.0
Sirt3/β-actin
*
Control siRNA
Sirt3 siRNA
Sirt3
β-actin
C
1.5
1.0
0.5
0.0
PINK1/β-actin
*
Control siRNA
PINK1 siRNA
PINK1
β-actin
